# Can Antioxidants Reduce the Toxicity of Bisphenol?

**DOI:** 10.3390/antiox11020413

**Published:** 2022-02-18

**Authors:** Wanda Mączka, Małgorzata Grabarczyk, Katarzyna Wińska

**Affiliations:** Department of Food Chemistry and Biocatalysis, Wrocław University of Environmental and Life Sciences, Norwida 25, 50-375 Wrocław, Poland

**Keywords:** bisphenol A, antioxidants, taurine, quercetin, geinstein, curcumin, lycopene, luteolin, naringin, melatonin

## Abstract

BPA is still the subject of extensive research due to its widespread use, despite its significant toxicity resulting not only from its negative impact on the endocrine system but also from disrupting the organism’s oxidative homeostasis. At the molecular level, bisphenol A (BPA) causes an increased production of ROS and hence a change in the redox balance, mitochondrial dysfunction, and modulation of cell signaling pathways. Importantly, these changes accumulate in animals and humans, and BPA toxicity may be aggravated by poor diet, metabolic disorders, and coexisting diseases. Accordingly, approaches using antioxidants to counteract the negative effects of BPA are being considered. The preliminary results that are described in this paper are promising, however, it should be emphasized that further studies are required to determine the optimal dosage and treatment regimen to counteract BPA toxicity. It also seems necessary to have a more holistic approach showing, on the one hand, the influence of BPA on the overall human metabolism and, on the other hand, the influence of antioxidants in doses that are acceptable with the diet on BPA toxicity. This is due in part to the fact that in many cases, the positive effect of antioxidants in in vitro studies is not confirmed by clinical studies. For this reason, further research into the molecular mechanisms of BPA activity is also recommended.

## 1. Introduction

**Figure 1:**
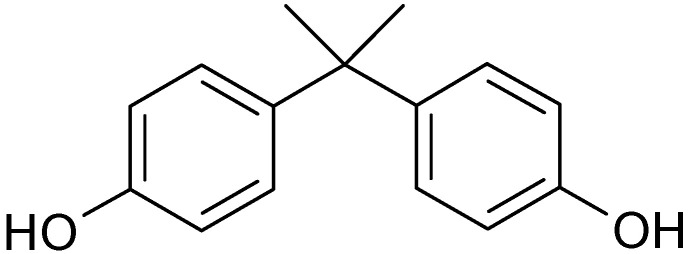
Chemical structure of BPA.

From a public health point of view, bisphenol A (BPA) is an important topic because it is commonly found in the human living environment. Global growth in BPA production (9618.7 kt by 2020) indicates that it is still popular with consumers [1]. BPA is 2,2-Bis-(4-hydroxyphenyl)propane (Figure 1), which was first synthesized by the Russian chemist A. P. Dyanin in 1891 by condensing two phenol molecules with one molecule of acetone in the presence of a strong acid or ion exchange resin containing acidic sulfonyl groups as a catalyst [2].

Bisphenol A is a white solid with a density of 1.2 g/cm^3,^ with a melting point of 158 °C and a boiling point of 360 °C. It is poorly soluble in water (120–300 ppm at 21.5 °C), while well soluble in organic solvents such as diethyl ether, acetone, benzene, ethanol, as well as in strong bases and acetic acid [3]. The lipophilic nature of BPA is also indicated by a logP value of 3.5. The half-life of BPA is about 4.5 days in water and soil, while in air it is less than one day, due to its low volatility [4,5].

BPA is used in many areas of industry, primarily to produce plastics such as polyacrylates, polyesters, polycarbonates, epoxy, and phenolic resins [6]. Polycarbonates are widely used due to their high durability in a wide temperature range (from −40 °C to +145 °C), hardness, resistance to acids, and transparency [7]. Both polycarbonates and epoxy resins are used in the manufacture of food storage packaging, e.g., beverage [8] bottles, baby bottles [9], and food storage containers [10]. Polycarbonates are also used for the manufacture of medical equipment [11], digital carriers such as compact discs [12], as well as toys for children [13]. Epoxy resins are used to coat cans containing food and beverages, as well as jar caps to protect food products from direct contact with metal [14]. In smaller quantities, BPA is also used in the production of thermal paper that is used in cash receipts [15], as well as an additive to products that are made of polyvinyl chloride (PVC) [16]. In turn, BPA-bisphenol A-glycidyl methacrylate (bis-GMA) is used in dental furniture and crowns. However, it can decompose or be contaminated with BPA and has, therefore, been found in the saliva of patients after a visit to the dentist [17].

Due to such different uses of bisphenol, its presence is often detected in wastewater, especially from factories that are engaged in industrial production [18,19,20]. It is also present in river sediments from rivers that are located in heavily urbanized areas [21,22]. Since BPA is present in waters, both fresh and salty [23,24,25,26], it is also absorbed by the organisms that are living in them. BPA is usually toxic to aquatic organisms [27,28,29]. However, some aquatic organisms, such as the Mediterranean mussel *Mytilus galloprovincialis* [30] and the Green-eared Mussel *Perna viridis* [31] or marine fish [32,33,34,35], are capable of accumulating BPA in the muscles and liver. Considering that both crustaceans and fish are often consumed by humans, the BPA that is contained in their meat enters our bodies also by increasing the level of exposure.

When studying BPA toxicity in rats after its oral administration, an LD_50_ of this compound was established at 3250 mg/kg body weight [36]. The reference dose of BPA for humans that was set by the US Environmental Protection Agency is 50 μg/kg·body weight and the acceptable estimated daily intake set by the European Food Safety Authority is 4 μg/kg·body weight.

BPA penetrates the skin because very high levels of BPA in plasma and urine have been found in cashiers in contact with the thermal paper of cash receipts. It can also cross the placental barrier and has been detected in the mother’s and foetal serum and placenta [37]. It has the ability to accumulate in various tissues, disrupting their physiological functions. It induces adipogenesis, stimulates lipid accumulation in adipose tissue and liver, and disrupts cytokine levels, thus contributing to the development of obesity, as well as hypertension and cardiovascular disease [38]. A link between BPA and the development of diabetes [39], anxiety, and depressive disorders [40], and an impaired functioning of the immune system has been found [41]. It can also contribute to infertility, endometriosis, and premature puberty [42,43]. However, BPA primarily exhibits hormone-like properties and, therefore, belongs to the class of endocrine disruptors. It binds easily to estrogen receptors (ERα and β), estrogen-bound gamma receptor (ERRγ), GPR30, androgen receptor, thyroid hormone receptors (TRα and β), and glucocorticoid receptors (GR) [37]. In addition, data that were obtained on cell lines show that BPA interferes with the synthesis, secretion, and signaling of thyroid hormones [44]. Due to its antiandrogenic activity, BPA acts as an estrogen receptor agonist and androgen receptor antagonist [37].

BPA has teratogenic, mutagenic, and carcinogenic effects, and can also interfere with the process of chromosome separation during meiosis. It can have a carcinogenic effect and increase susceptibility to cancer [7]. Extensive research over the past decade has shown a close relationship between BPA exposure and the occurrence of various cancers, especially breast and ovarian cancers. The effect of BPA on the development of tumors is primarily related to its hormone-like effect. However, a growing body of evidence indicates that the induction of reactive oxygen species (ROS) by BPA is a significant factor influencing its toxicity and carcinogenic potential. Therefore, the question arises whether antioxidants can reduce BPA toxicity and, above all, reduce the risk of cancer induction. In the following study we will try to answer this question, especially since many antioxidants not only have a positive effect on cellular antioxidant mechanisms but also have anti-cancer activity.

## 2. Metabolism of BPA

**Figure 2:**
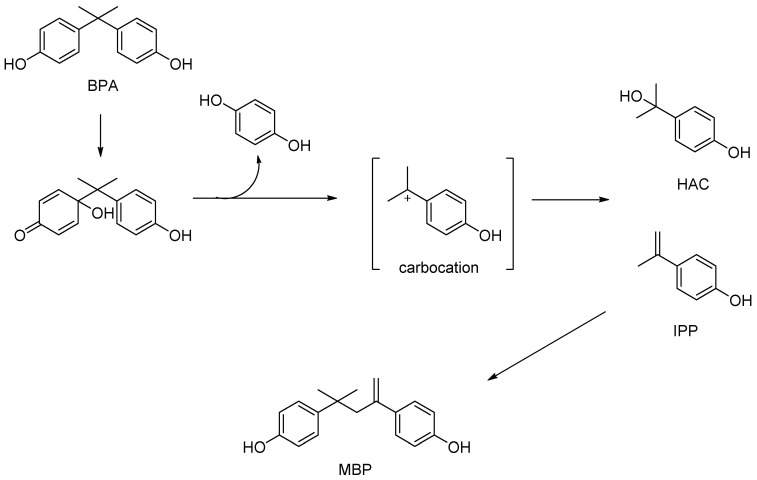
Suggested mechanism of formation the three estrogenic metabolites of BPA.

The metabolism of BPA has been extensively studied using both in vivo and in vitro systems. This compound is present in human serum, urine, amniotic fluid, and breast milk in populations of industrialized countries around the world [45]. Orally-introduced BPA must pass through the digestive tract and liver before reaching the uterus or testicles. Due to the presence of hydroxyl groups, BPA is mainly directly coupled by uridine 5′-diphospho-glucuronosyltransferase (UGT) to BPA mono-glucuronide [46]. It is less strongly conjugated with sulfuric acid by cytosolic sulfotransferase [47]. Thus, it does not require prior activation by cytochrome P450 (CYP) monoxygenases. However, CYP can metabolize BPA to bisphenol-o-quinone mediated by 5-hydroxy BPA and the bisphenol-semiquinone intermediate [48,49]. In a study by Nakamura et al. [50], CYP3A4 and CYP3A5 were shown to have the ability to cleave BPA via ipso substitution, forming two metabolites isopropenylphenyl (PPI) and hydroxy cumulative alcohol (HAC). It is worth noting that both metabolites can be bound by estrogen receptors (Figure 2). BPA was also incubated with the S9 fraction of human, monkey, rat, and mouse liver [51]. The metabolite, 4-Methyl-2,4-bis(p-hydroxyphenyl)pent-1-ene (MBP) was isolated and it also showed several times higher estrogenicity than BPA [52]. In addition, BPA may adversely affect the hepatic metabolism of other xenobiotics, including drugs and it can inhibit the activity of such CYP isoforms as CYP1A2, CYP2A2, CYP2B2, CYP2C11, CYP2D1, CYP2E1, and CYP3A2. BPA competitively inhibits the 17α-hydroxylation of progesterone by human CYP17 [53]; non-competitively inhibits CYP2C8, which catalyzed aminopyrine N-demethylation; and shows a mixed type of inhibition of S-mephenytoin 4-hydroxylation by CYP2C19 [54].

BPA can be coupled with sulfuric acid via the phenol sulfotransferase SULT1A1 and ST1A3 [55,56]. However, the most important reaction is glucuronic acid conjugation with BPA, which is catalyzed by UDP-glucuronosyl transferase. BPA is highly glucuronidated as it passes through the rat intestinal lumen [57]. Analyses that were performed on a mouse cell line in which all UGT2B genes were deleted suggest that members of the UGT1 family play an important role in intestinal BPA glucuronidation [58]. In turn, in the liver, the glucuronidation of BPA is mediated by UGT2B1, an isoform that glucuronidates some endogenous androgens and is not expressed in the rat intestine [59]. In humans, several isoforms of UGT are known to couple with BPA. UGT2B15 has the highest activity, with less activity being noted for recombinant UGT1A1, UGT1A3, UGT1A9, UGT2B4, and UGT2B7 [60].

## 3. Preservation of Oxidative Homeostasis in the Organism

Every cell in the human body maintains a state of homeostasis between oxidizing agents (e.g., reactive oxygen species (ROS), reactive nitrogen species (RNS)), and antioxidants. Members of the reactive oxygen species that are present in human body include singlet oxygen, superoxide anion (O_2_^●−^), hydrogen peroxide (H_2_O_2_), peroxyl radical (ROO^●^), and hydroxyl radical (^●^OH). In turn, nitrogen free radicals include nitric oxide (NO^●^) and peroxynitrite anion (ONOO^●^) [61]. Up to 1–3% of the oxygen that is taken up by the lungs is converted into ROS. Under normal metabolism, the continuous formation of ROS and other free radicals is important for normal physiological functions such as ATP production, various catabolic and anabolic processes, and cellular redox cycles [62]. Oxidative stress is characterized by a critical imbalance between the antioxidant defense and the free radicals [63,64]. It occurs when homeostatic processes fail and the production of free radicals far exceeds the body’s defences, promoting cell and tissue damage. These damages can include DNA, proteins in cells, peroxidation of cell membrane lipids, calcium influx, and mitochondrial edema and lysis [62]. Antioxidants scavenge and neutralize the free radicals, thus preventing oxidation reactions and restoring cell integrity. In general, they can be divided into two groups: enzymatic antioxidants (including superoxide dismutase (SOD, EC 1.15.1.1), catalase (CAT EC 1.11.1.6), glutathione S-transferase (GST, EC 2.5.1.18), glutathione peroxidase (GPx, EC 1.11.1.9)), and non-enzymatic antioxidants. Enzymatic antioxidants are capable of gradually converting oxidized transformation products. They break down the superoxide radical, hydrogen peroxide (H_2_O_2_), and hydroperoxides, respectively, into harmless molecules (water/alcohol and oxygen). Non-enzymatic antioxidants such as vitamins A, E, C, flavonoids, carotenoids, glutathione, and melatonin, interrupt and terminate the free radical chain reactions. It is worth emphasizing that antioxidants can safely interact with the free radicals that are generated by exposure to BPA and interrupt the chain oxidation reaction before important molecules such as DNA, RNA and proteins are damaged [65].

## 4. BPA and Enzymatic Antioxidants

In animal models, BPA severely damages the function of many vital organs and cells, such as liver, kidney, pancreas, and testes, what is accompanied by an increased production of ROS. In addition, BPA disturbs the dynamic balance of enzyme antioxidants, reducing the levels of SOD, GSH, and CAT in the blood and pancreas [65].

Superoxide dismutase (SOD, EC 1.15.1.1) is a metalloenzyme that is the first line of defense against harmful ROS. It catalyzes the dismutation of two molecules of the superoxide anion (O_2_^●−^) to hydrogen peroxide and molecular oxygen, making the potentially harmful peroxide anion less dangerous [64]. There are three isoforms of SOD in humans: cytosolic copper/zinc-dependent Cu/Zn-SOD (or SOD1), mitochondrial manganese-dependent Mn-SOD (or SOD2), and extracellular Cu/Zn-dependent EC-SOD (or SOD3) [66]. SOD2 is the major antioxidant enzyme that scavenges ROS (especially superoxide) in the inner mitochondrial matrix and acts as the first line of defense against mitochondrial oxidative damage. BPA influences the expression of SOD2, which is important as this enzyme is extremely important in regulating the mitochondrial death pathway and signaling apoptosis. Therefore, the altered expression of this enzyme under BPA exposure may be directly related to abnormal target cell growth and differentiation [67]. On the other hand, when mice were administered with BPA intraperitoneally at doses of 25 and 50 mg/kg/day, increased SOD activity was observed [68]. In contrast, no significant changes in SOD levels were noted in studies of neonatal mouse ovary cells after in utero or in vitro exposure to BPA. This may be due to the fact that the applied doses of BPA (50 μg/kg body weight) were not sufficient to induce and/or weaken the antioxidant defense in mice. Therefore, further studies seem necessary, especially dose-dependent studies, to determine the appropriate BPA levels that guarantee optimal SOD activity in different cells [65].

Another important enzyme in the body’s antioxidant defense is catalase (CAT, EC 1.11.1.6), which is found in almost all aerobic organisms [69]. In animals, CAT is present in every organ. However, its particularly high levels are found in the liver. The enzyme uses iron or manganese as a cofactor. Human catalase, having a molecular weight of about 220–240 kDa, contains protoporphyrin IX as a prosthetic group [70] and catalyzes the decomposition or reduction of hydrogen peroxide to water and molecular oxygen, consequently terminating the detoxification process that is initiated by SOD [64].

CAT prevents the oxidative action of destroying 17β-estradiol and diethylstilbestrol. Banerjee et al. [71] evaluated the effects of nine-day intraperitoneal administration of BPA to sexually mature female rats. The exposure to BPA significantly increased the level of nitric oxide and lipid peroxidation in the ovarian granular cells, a marked decrease in the level of estrogen and progesterone, an increase in the activity of proinflammatory cytokines (tumor necrosis factor α and interleukin 6), and significantly decreased CAT expression. In addition, administration of the CAT-specific blocker, 3-amino-1,2,4-triazole (ATZ; 1 g/kg body weight/day for 5 days, ip), to the female mice with BPA enhanced these effects. Therefore, CAT plays a major role in the functional integrity of the ovarian granulosa cells and the reproductive capacity of women under stress, a process which is disrupted by BPA [71].

Kabuto et al. found that five-day exposure of male mice to 25 and 50 mg BPA/kg/d significantly decreased the activity of CAT in the liver. However, the authors did not investigate whether the altered CAT activity was responsible for the functional changes in the liver cells [68].

Glutathione (GSH) is a water-soluble tripeptide consisting of glutamine, cysteine, and glycine. GSH plays a role in the detoxification of various electrophilic compounds and peroxides by participating in enzymatic reactions that are catalyzed by glutathione S-transferases (GST) and glutathione peroxidases (GPx) [72]. GST is one of the major phase II detoxifying enzymes and plays a pivotal role in detoxifying and excreting toxins in the liver by coupling them to GSH [73]. GPx, on the other hand, is a selenoenzyme that catalyzes the reduction of hydrogen peroxide to water using glutathione (GSH) and can, therefore, prevent oxidative damage to mammalian cells [74].

The intraperitoneal administration of BPA (50 mg/kg/d) for five days to adult male mice increased the level of GSH + GSSG in the brain, kidneys, liver, and testes, while in the testes there was a decrease in GSH activity. The results that were obtained suggest that the injection of BPA induced an overproduction of hydrogen peroxide in mice. Hydrogen peroxide is easily converted into a hydroxyl radical. The decrease in GSH and the increase in GSSG may be caused by the hydroxyl radical. BPA can show its toxicity by increasing the level of hydrogen peroxide [68].

Pregnancy exposure to BPA (50 mg/kg body weight/day) significantly reduces the expression of GSH S-transferases in sperm from adult F1 male mice. Reduced levels of GSH S-transferase are associated with a loss of fertility in male offspring. In other studies, a decreased expression of GSH S-transferases and an increased expression of GSH peroxidase were observed in mature mouse sperm after in vitro exposure to 1 μM BPA for six hours. The altered antioxidant activity in sperm was also associated with an abnormal acrosomal response and impaired the detoxification process of the mitochondria, subsequently affecting the ability of the sperm to fertilize the oocyte in the in vitro fertilization test [65].

When BPA was administered to rats in an amount of 130 mg/kg bw that was dissolved in olive oil, decreased activity of catalase (CAT), glutathione S-transferase (GST), glutathione peroxidase (GPx), and superoxide dismutase (SOD) was observed. Additionally, the biochemical changes were accompanied by histopathological changes indicating the destruction of the normal structure of the liver. Histological studies have shown that exposure to BPA causes sinus dilatation, inflammatory cell infiltration, congestion, and necrosis of the liver parenchyma [75].

## 5. Survey of Antioxidants That Affected BPA Toxicity

### 5.1. Genistein

Genistein is an isoflavone (5,7-dihydroxy-3-(4-hydroxyphenyl)chromen-4-one), the main source of which is legumes, especially soybeans. It is also present in broad beans, chickpeas, vegetables, fruits, and nuts [76]. It is worth noting that genistein, which is an aglycone of genistin, is present in soy products because genistin is dominant in unprocessed soybeans [77]. Additionally, genistein is released from glycosides by the intestinal enzyme glucosidase [78]. Genistein has good affinity for the β-estrogen receptor, where the 4-OH group interacts with the side chains of Glu305, Arg346, and the water molecule to mimic the A ring of estradiol, while the 7-OH group of the flavone core is hydrogen-bonded to His475 at the distal end of the binding cavity [79]. It also binds to α-receptors. For this reason, genistein helps in the regulation and treatment of various disorders that are related to estrogen hypo- and hypersecretion. It also demonstrates the ability to scavenge reactive oxygen species (ROS) and reactive nitrogen species (RNS) [77]. It prevents lipid peroxidation, strengthens the antioxidant system, and reduces liver damage that is caused by CCl_4_ [80]. It also has anti-cancer properties. In studies that were carried out on cell lines of such cancers as colorectal cancer (HCT-116, LoVo [81], HT-29, [82,83]), pancreatic cancer (Mia-PaCa2, PANC-1) [84], human breast cancer (MCF-7, MDA-MB-231 [85], SK-BR-3 [86] and ZR-75-1 [87]), esophageal cancer (Eca-109, EC9706 and CaES-17) [88], and prostate cancer (PC3) [89], genistein has been shown to be a potent inhibitor of cancer cell proliferation. In contrast, the role of genistein in the proliferation of breast cancer cells depends mainly on genistein dose, exposure time, and the relative α/β ratio of nuclear estrogen receptors (ER). The results of clinical trials showed that dietary genistein did not adversely affect either the proliferation of breast cells or the thickness of the endometrium [90]. Genistein exerts its antitumor activity through its pleiotropic molecular mechanism, acting on the cell cycle, apoptosis, angiogenesis, invasion, and metastasis [91].

In the studies of Jadhav et al. [92] an attempt was made to explain why exposure to endogenous hormones and environmental chemicals early in development can cause long-term changes that can lead to the development of breast cancer. DNA methylation studies revealed that exposure to BPA early in life alters signaling pathways that contribute to carcinogenesis. However, genistein reduces or counteracts some of the changes that are caused by the carcinogenic properties of BPA. In immature rats that were exposed to 250 μg/kg/day BPA (postnatal days 2–21), most of the genes were hypermethylated. A total of 12 genes were identified that were homologous to human genes that are associated with the survival rate of ER-positive breast cancer patients. There were two genes in particular: HPSE and RPS9 that were hypomethylated in the mammary glands of rats that were exposed to genistein or genistein + BPA, respectively, suggesting the suppressive properties of this phytoestrogen [92,93].

In turn, studies of the influence of genistein and BPA on the metabolism of estrone (E1) in rat liver microsomes showed that both substances inhibited 2-hydroxylation and 16α-hydroxylation of E1, but to a different extent, thus reducing the 2-OH-E1/16α ratio, what is biomarker of breast cancer risk. Although the 2-OH-E1/16α-OH-E1 ratio was lower in the BPA-treated group than under control conditions, the difference was not statistically significant. The 2-OH-E1/16α-OH-E1 ratio was significantly reduced in the genistein group (*p* < 0.05), and this effect was more pronounced with the combination of BPA and genistein (*p* < 0.01). The results suggest a synergistic effect of genistein and BPA on E1 metabolism, which is associated with an increased risk of breast cancer development [94].

A study in laying hens investigated the effects of BPA and genistein at reference doses on adult ovaries. BPA significantly reduces the production efficiency and weakens the ovarian redox homeostasis by activating Keap1 and suppressing the Nrf2-signaling pathway (Nrf2, NQO1, and HO-1). On the other hand, genistein at a dose of 20, 40 mg/kg bw/day enhanced the activity of antioxidant enzymes CAT, GPx, and SOD; improved the functioning of the Nrf2-Keap1 pathway; and inhibited an excessive accumulation of lipid peroxidation products (MDA). Additionally, exposure to BPA and genistein alters the expression of the estrogen receptor alpha (ERα) in the ovary. The use of a specific ERα agonist/antagonist confirmed that BPA and genistein have opposite regulatory effects on the Nrf2-Keap1 pathway by targeting Erα [95].

In turn, the influence of BPA and genistein on the secretion of cytokines and chemokines was investigated using PMA-differentiated-U937 (a widely used cell line for primary human macrophages). BPA suppressed the cytokine/chemokine secretion at 1 mM while increased their secretion at 10 mM. Genistein reversed the macrophage inhibitory effects of BPA at high doses and produced synergistic effects with BPA at low concentrations [96].

In a further study, pregnant Sprague-Dawley rats were dosed with BPA at 25 or 250 mg/kg/day by gavage from day 10 to 21 of gestation with or without genistein at 250 mg/kg/food (~5.5 mg/kg/day). The exposure to BPA induced early prostate changes, while genistein attenuated some of the deleterious effects of this xenoestrogen on the level of epithelial cell proliferation, androgen receptor expression, and prostate architecture in male offspring. At PND 180, a significant increase in the incidence of multifocal prostatitis/reactive hyperplasia and atypical hyperplasia was observed in male offspring from mothers receiving BPA 25 mg/kg/day. On the other hand, genistein alleviated some of the adverse effects of BPA pregnancy exposure in early and late prostate development in F1 offspring [97].

ERβ expression was also found in B and T lymphocytes. Antiproliferative activity of BPA (50 µg/kg BW/d) and genistein (1 mg/kg BW/d) on the growth of mouse (EG7) and human lymphoma (Granta-519) was found in vivo in a mouse model. Researchers even suggest the use of dietary supplements with phytoestrogens in the prevention of lymphoid tumors [98].

### 5.2. Curcumin

Curcumin (1,7-bis(4-hydroxy-3-methoxyphenyl)-1,6-heptadiene-3,5-dione) is also called diferuloylmethane. Curcumin is the main natural polyphenol that is found in the rhizomes of *Curcuma longa* and other *Curcuma* species. *Curcuma longa* has been used traditionally in Asian countries as a medicinal herb. Curcumin has antioxidant properties as it increases the activity of SOD and CAT. It can also inhibit ROS-producing enzymes such as lipoxygenase/cyclooxygenase and xanthine hydrogenase/oxidase [99]. Moreover, it inhibits the formation of hydroxyl radicals and superoxide anions by preventing the oxidation of Fe^2+^ to Fe^3+^ in the Fenton reaction [100]. Curcumin may reduce heavy metal-, chemical-, and drug-induced hepatotoxicity. It prevents lipid peroxidation and histological damage to the liver, demonstrating anti-inflammatory and antioxidant effects, and protects against mitochondrial dysfunction. In turn, the anti-cancer properties of curcumin have been described in many review articles, e.g., [101,102,103,104].

In a study by Apaydin et al. [105] in rats that were administered BPA only, pathological changes in the small intestine such as necrosis or degenerative changes of the villus that may be caused by oxidative damage of BPA in intestinal cells were observed. Due to the high antioxidant potential of curcumin, the beneficial effects of the combination of curcumin dissolved in olive oil in the amount of 100 mg/kg bw with BPA (130 mg/kg bw) were expected. However, it has not been shown that curcumin can fully protect an organism against BPA toxicity [105].

In turn, in the studies that were conducted by Uzunhisarcikli et al., rats were administered the same dose of curcumin and BPA as in the above study. The treatment with curcumin significantly alleviated the oxidative stress that was induced by BPA, mainly through the preservation of endogenous liver antioxidants such as GPx, GST, CAT, and SOD. In addition, curcumin itself neutralizes ROS and activates the transcription factor Nrf2, which is responsible for the regulation of the expression of genes that encode most of the phase II detoxification enzymes and antioxidant enzymes [106]. Moreover, no necrosis was observed in the liver tissues of rats that were administered curcumin together with BPA [75].

### 5.3. Lycopene

Lycopene is mainly found in tomatoes and is an example of a carotenoid with a linear structure containing eleven conjugated and two unconjugated double bonds [107]. The structure of the conjugated polyene is responsible for the ruby colour and the antioxidant properties of lycopene [108]. It is the most effective in vitro free radical scavenger of all carotenoids [109]. For example, it can remove two and ten times more singlet oxygen than beta-carotene and alpha-tocopherol, respectively [108]. Lycopene protects the cell membranes and DNA strands against oxidative damage and reduces the structural changes that are caused by free radicals [110]. However, the double bonds in the structure of this compound can readily be isomerized from all trans- to mono- or poly-cis forms by exposure to light, temperature, or chemical reactions. Therefore, about 90% of the lycopene that is found in tomatoes is completely trans-isomer, while more cis-isomers are found in processed foods [111]. Lycopene is lipophilic in nature, which makes it almost insoluble in ethanol, methanol, and water. It is not provitamin A and is not synthesized by man. The results of clinical trials confirm its protective effect on the cardiovascular system as it lowers the risk of myocardial infarction and lowers the blood pressure. High levels of lycopene in the blood are also associated with a lower risk of developing prostate, lung, uterine, and breast cancer. Lycopene not only inhibits the proliferation of cancer cells, but also induces their apoptosis and prevents metastasis [108]. It enhances the expression of pro-apoptotic genes, including caspase 3, 9, and p53 [112].

According to Rao et al. [113], the daily consumption of lycopene to provide a beneficial effect on the human body should be from 5 to 7 mg/day. However, in the case of cancer, the daily recommended dose is increased to 75 mg [114]. In a study by Mohammed et al. [115], it was shown that lycopene, when it was administered concurrently with BPA, caused significant improvements in the health of the liver, spleen, and pancreas at the structural and ultrastructural levels.

In the lungs, BPA caused a chronic inflammatory response in the form of increased macrophages, lymphocytic infiltration, congestion, and interstitial haemorrhage and fibrosis. Increased levels of pro-inflammatory interleukins IL-1β and IL-6 have been found, in addition to decreased levels of anti-inflammatory IL-10. Additionally, BPA causes a significant increase in Bax and caspase-3 expression and a decrease in Bcl-2 expression. Lycopene mitigated all the toxic effects of BPA and brought all the oxidant/antioxidant markers back to near-normal levels. It caused an increase in GSH level and SOD activity. Significantly increased expression of Bcl-2 and decreased expression of Bax and caspase-3 were observed in the group of rats that received lycopene and BPA compared to the BPA group. The anti-apoptotic properties of lycopene can be explained by the inhibition of the caspase-3 pathway and Bax expression [116]. This action of lycopene protects the heart tissue from the toxicity of diclofenac sodium and tulathromycin [117].

In a study with female Wistar rats, lycopene 10 mg/kg body weight and BPA 10 mg/kg body weight were administered daily by gavage for 30 days. In rats that were administered BPA alone, high levels of serum liver enzymes were found with low levels of total proteins (TP) and albumin. BPA also induced hepatic oxidative stress. The administration of lycopene reduced the cytotoxic effect of BPA on hepatic tissue by improving the biomarkers of liver function and the oxidative-antioxidant state, as well as DNA damage around the control values. The beneficial antioxidant effect of lycopene was confirmed by the increased antioxidant activity of SOD, GPx, and CYP along with decreased levels of malonyldialdehyde (MDA) in rats that were co-administered with BPA and lycopene. Lycopene protects against the oxidation of proteins, lipids, and DNA by scavenging singlet oxygen and superoxide radicals, thus limiting the production of MDA as an end-product of lipid peroxidation [118].

In another study, pregnant mice were administered BPA orally at 500 mg/kg/day from day 8 of gestation to day 14. The second group was administered lycopene at a dose of 20 mg/kg/day from days 1 to 7, followed by BPA at a dose of 500 mg/kg/day from 8 to 14 day. BPA lowered testosterone, luteinizing hormone, and follicle-stimulating hormone, while lycopene treatment increased them significantly. BPA raised estradiol, while lycopene lowered estradiol in the offspring. BPA caused testicular damage, as indicated by fewer Leydig cells and ovarian damage, less body granularity in adult offspring, while lycopene reduced the damage. The mother’s exposure to BPA increased Bax and decreased Bcl-2 in testicular and ovarian tissue in the offspring of mice. Lycopene decreased Bax in the testes and ovaries, and increased Bcl-2 in the ovarian tissue in the offspring of mice. Lycopene, therefore, has a protective effect on the reproductive toxicity that is caused by exposure to BPA [119].

### 5.4. Luteolin

Luteolin is 3′,4′,5,7-tetrahydroxyflavone (2-(3,4-dihydroxyphenyl)-5,7-dihydroxy-4*H*-1-benzopyran-4-one) and is naturally found in many fruits, vegetables, and medicinal herbs. This compound is insoluble in cold water and slightly soluble in hot water [120]. It is often present as glycosides, which can be hydrolyzed by lactase-phlorizin hydrolase (LPH) and enterobacteria, or is absorbed by the sodium-glucose co-transporter 1 (SGLT1) on the surface of the intestinal cells [121]. Luteolin is metabolized into glucuronide or sulfate conjugates [122]. It has been shown to have strong anti-inflammatory, anti-diabetic, hepatoprotective, anti-cancer, anti-apoptotic, and chemoprotective properties [123].

In studies that were carried out on *Drosophila melanogaster*, BPA was shown to induce antioxidant-oxidative imbalance and a behavioral deficit. Luteolin that was administered at a dose of 50, 100, 150, and 300 mg/kg increased *D. melanogaster* survival by 58, 68, 68, and 73%, respectively. Luteolin increased the activity of GST, CAT, and acetylcholinesterase (AChE). However, while luteolin improved the catalase activity, it did not prevent the BPA-induced increase in H_2_O_2_ levels. In addition, thiol-containing compounds act as reducing agents to maintain cellular redox homeostasis. The fact that luteolin increased the total thiol levels and prevented BPA-induced thiol depletion indicates its contribution to maintaining the redox balance despite the presence of toxic BPA metabolites [124].

The nephroprotective effect of luteolin on BPA-induced toxicity was investigated in rats that were dosed orally at 100 and 200 mg/kg. Luteolin reduced BPA-induced kidney abnormalities, blood urea nitrogen, serum creatinine, and serum uric acid. Additionally, it was observed that luteolin decreased the production of such inflammatory mediators as TNF-α, IL-1β, and IL-6 and enhanced the expression of Nrf2 and heme oxygenase 1 (HO-1). Luteolin activates the Nrf2/ARE/HO-1 defense pathway, which plays an important role in the therapeutic treatment of BPA-induced renal toxicity [125].

### 5.5. Melatonin

Melatonin (N-acetyl-5-methoxytryptamine) is an important endogenous hormone that is secreted mainly by the pineal gland that regulates the circadian rhythm and is involved in the maintenance of redox homeostasis. Due to its small size and lipophilicity, this compound easily crosses biological membranes and reaches all cell compartments. Melatonin directly enhances the activity of NK cells, stimulates the secretion of IL-2 and IL-6, and protects hematopoietic progenitor cells against radiotherapy and chemotherapy [126]. Melatonin and its metabolites can directly scavenge various free radicals. It also reduces oxidative stress by stimulating the activity of SOD and GPx [127]. It is worth noting that melatonin has been found to have an oncostatic effect, especially in estrogen receptor-positive breast cancer. The suppression of ER mRNA expression and ER transcriptional activity through the MT1 receptor was observed [128]. Melatonin also regulates the transactivation of nuclear receptors, estrogen metabolizing enzymes, and the expression of related genes. Additionally, melatonin inhibits tumor oxygen glycolysis and critical cell signaling pathways that are essential for cell proliferation and metastasis formation. Importantly, melatonin also abolishes resistance to hormone therapy and chemotherapy [126].

The effect of melatonin on the regulation of the transcription factor OCT4 (Octamer Binding 4) by the alpha estrogen receptor (ERα) in breast cancer stem cells (BCSC) was investigated. The cells were treated with 10 nM estrogen (E2) or 10 µM BPA and 1 mM melatonin. E2 or BPA induced increases in the number and size of mammoths that were reduced by treatment with melatonin. In addition, the binding of ERα to OCT4 was decreased, accompanied by a reduction in OCT4 and ERα expression [129].

Subsequent studies were performed in Sprague-Dawley rats that were administered BPA at a dose of 200 mg/kg body weight per day for ten consecutive days. BPA caused a decrease in the SOD activity, which eased pretreatment with melatonin, which was administered at a dose of 10 mg/kg body weight/day [130].

In turn, in a study on MCF-7 and T47D breast cancer cell lines, melatonin (1 nM) significantly reduced cell proliferation that was raised by BPA (100 nM). It also lowered the activity of phosphorylated extracellular regulated kinase (ERK) and serine/threonine specific protein kinase (AKT). The concurrent administration of BPA and melatonin also increased the levels of melatonin receptor 1 (MT1). Melatonin can also significantly block the increased expression of steroid receptor coactivators (SRC-1, SRC-3) and the activity of the BPA-induced estrogen response element [131,132].

The beneficial effects of melatonin in alleviating BPA-induced oxidative stress were also found in a subsequent study when Sprague Dawley rats were administered subcutaneously with BPA (100 μg/kg BW, 1 mg/kg BW and 10 mg/kg BW) and melatonin (4 mg/kg BW) for 16 days. Exposure to BPA caused oxidative stress and further increased the expression of PUMA, p53, and Drp-1, causing apoptosis in rat brain tissue. Melatonin significantly attenuated the toxic effects that were induced by BPA by scavenging the ROS, increasing the activity of antioxidant enzymes (SOD, CAT, ascorbate peroxidase (APX), Px), and attenuated brain apoptosis by normalizing p53, PUMA, and Drp-1 expression both at the transcriptional and translational levels. Additionally, histological studies showed normalization of the cellular architecture by melatonin [133].

In the studies of Olukole et al. [134], it has been shown that a low dose of melatonin can protect against BPA-induced prostate toxicity. Adult Wistar rats were administered intraperitoneally 10 mg/kg bw/day of melatonin and orally BPA that was dissolved in DMSO and dissolved in rapeseed oil at a dose of 10 mg/kg BW/day for 14 days. BPA significantly increased the rat prostate index, while melatonin reduced it. BPA significantly increased the serum estrogen levels as well as prostate-specific antigen, but decreased serum testosterone levels in rats, while concomitant administration of melatonin attenuated these changes. BPA also caused vascular hyperplasia, prostatic epithelial hyperplasia (functional, reactive, and atypical), and tubular atrophy in rats, while melatonin attenuated the observed changes [134].

The effect of *Ginkgo biloba* extract, melatonin, and their combination against BPA-induced liver and kidney toxicity in male rats was also investigated. Elevated plasma levels of TNF-α and ALT and AST activity in combination with increased urea and creatinine levels with a concomitant reduction in total plasma protein may reflect the deleterious effect of BPA. Protective effects against BPA-induced hepato- and nephrotoxicity were observed when the BPA-treated rats were concurrently administered *G. biloba* extract, melatonin, or a combination thereof. Both the *G. biloba* extract and melatonin increased the level of GSH and the activity of SOD, GST, and CAT [132,135].

### 5.6. Naringin

Naringin is a flavan-7-*O*-glycoside between the naringenin flavanone and the rhamnose disaccharide. This glycoside is found mainly in citrus fruits, especially grapefruits. It has a distinct bitter taste and is moderately soluble in water [136]. The gut microflora breaks down naringin into its aglycone naringenin, which is then absorbed and metabolized in the liver by conjugation with sulfuric and glucuronic acid [137]. It has antioxidant properties; however, it is weaker than its aglycone naringenin because the sugar moiety causes a steric obstacle in scavenging free radicals. Naringenin in its structure has numerous double bonds that reduce the energy necessary for electron delocalization, providing a good source of hydrogen atom donation for stable free radicals. Naringenin readily crosses the blood-brain barrier and significantly reduces lipid peroxidation, restoring the proper activity of SOD, GST, and GPx [138]. Naringin significantly inhibits the activity of xanthine oxidase in vitro, which is a physiological source of superoxide anions in eukaryotic cells [139]. It normalizes the elevated concentrations of TNF-α in inflammation and the infiltration of inflammatory cells [140]. In a study in rats with Walker carcinoma, naringin (25 mg/kg bw) inhibited the tumor growth by 75% by reducing the levels of IL-6 and TNF-α [141]. Additionally, elevated levels of TNF-α and IL-1β were observed in human monocytic leukemia (THP-1) cells after exposure to BPA (0.1–10 μM) [142]. It also inhibits the expression of NF-κB, cyclooxygenase-2, and cytokines. Narigin-supplementation may help prevent obesity, high blood pressure, and diabetes [136]. It also shows hepatoprotective activity, because supplementation with naringin lowered the increased nickel-induced plasma transaminase activity in rats (naringin: 80 mg/kg bw) [143]. Naringin blocks the synthesis of TGF-β by inhibiting MMP-13 in liver damage [144]. TGF-β is a protein that is involved in many cellular processes including differentiation, growth, carcinogenesis, angiogenesis, and extracellular matrix formation [145].

In studies by Akintude et al. [146] in albino rats with hypertension that was induced by the arginine analogue (L-NAME, Nω-nitro-L-arginine methyl ester hydrochloride), it has been shown that naringin supplementation (80 mg/kg) can reduce the toxicity of BPA (50 mg/kg). Naringin was observed to prevent the reduction in nitric oxide (NO) levels. NO is one of the critical mediators of inflammation in humans as well as in murine cancer models. NO is produced by induced nitric oxide synthase/nitric oxide synthase 2 (iNOS/NOS2) in vascular endothelial cells and on the one hand dilates blood vessels and on the other hand participates in the immune response to cancer and infection [147]. NO activates guanylate cyclase resulting in an increased production of cyclic guanosine monophosphate (cGMP).

Naringin inhibits the expression of TNF-α and IL-1β, the expression of which is increased by BPA [146,148]. They are pro-inflammatory cytokines that are secreted by macrophages of the M1 type and are involved in the regulation and expression of various genes that play key roles in oncogenesis and the inflammatory process. BPA significantly increases caspace-9 and p53 levels, and naringin reduces caspace-9 expression. Naringin prevented the reduction of NO by lowering the levels of caspace-9 and p53 proteins. The p53 protein regulates the cell cycle, acts as a tumor suppressor, and is undetectable in healthy tissue. The transcription of the p53-encoding gene is strongly increased in tumor cells or tissues after ischemic injury [146]. Naringin lowered the level of CD43, which is present in over 90% of T-cell lymphomas and in B-cell lymphoblastic lymphoma. Since it stains granulocytes and their precursors, it is also an effective marker of myeloid tumors. The reduction of apoptotic proteins (caspace-9 and p53) caused cGMP self-stimulation during smooth muscle relaxation [148].

Naringin may be protective against BPA-induced cardiotoxicity in Wistar rats that were administered 50 mg/kg of BPA and 40, 80, and 160 mg/kg of naringin for 30 days. In rats that were administered BPA alone in a dose of 160 mg/kg, a significant increase in the levels of aspartate aminotransferase, lactate dehydrogenase, creatine kinase-MB, triglycerides, lipid peroxidation, and a significant decrease in glutathione, superoxide dismutase, catalase, and glutathione peroxidase were observed. Naringin at a dose of 80 and 160 mg/kg was protective against BPA-induced cardiotoxicity due to its antioxidant activity and inhibition of lipid peroxidation [149].

It was also investigated whether naringin (40, 80, and 160 mg/kg/day) that was administered simultaneously with BPA (50 mg/kg/day) could modify the influence of BPA on cognitive functions and inhibit possible oxidative stress in the brain tissue of rats. Naringin administration led to BPA restoring the depleted GSH and GPx activity, suggesting the ability of naringin to enhance oxidative defense by restoring the levels of these antioxidants. At the cellular level, naringin alleviates the toxic effects of reactive oxygen species by directly stimulating the transcription of genes encoding several antioxidant enzymes such as CAT, SOD, and GPx. This may explain the fact that administration of naringin to BPA-treated rats caused a significant increase in CAT and SOD activity. The administration of naringin also attenuated AChE activity after BPA administration. BPA is known to reduce the activity of acetylcholine transferase, which causes cognitive dysfunction and oxidative stress. Naringin also lowered the concentration of malondialdehyde and nitrite, which were increased by BPA [150].

Another study by the same group of researchers showed that BPA significantly increased the levels of triglycerides, lactate dehydrogenase (LDH), alkaline phosphatase (ALP), lipid peroxidation, and aspartate aminotransferase (AST), and significantly decreased the activity of CAT, GPx, SOD, and glutathione. In addition, BPA caused periportal inflammation and microbubal steatosis in rat tissue. The administration of 80 and 160 mg/kg of naringin reduced the BPA-induced hepatotoxicity and changed the level of lipid and liver enzyme peroxidation in rat blood serum [151].

### 5.7. Quercetin

Quercetin is 3,5,7,3′,4′-pentahydroxyflavone that is commonly found in many vegetables and fruits [152]. The antioxidant potential of quercetin is approximately four times greater than that of vitamin E [153]. In addition, quercetin supplementation inhibits the expression of IL-1β, IL-6, NF-κB, iNOS, and TNF-α proteins and mRNA. It is worth noting that NF-κB is a major transcriptional mediator that plays a key role in the cell’s response to a diverse set of inflammatory stimuli and mediates the expression of over 500 genes, including IL-6, IL-1β, TNF-α, and iNOS [154]. The anti-tumor activity of quercetin has recently been reported in several review articles [155,156,157,158]. It also exhibits antihypertensive, vasodilator, anti-obesity, anti-hypercholesterolaemic, and anti-atherosclerotic activities [65].

Quercetin has an anti-estrogenic effect and increases the testosterone levels in male rats [159]. It also reverses toxic histological and biochemical changes and reduces the genotoxic effects of BPA. It reduces the rate of apoptosis that is associated with DNA fragmentation [160]. With the concomitant administration of quercetin and BPA, it has been observed that this flavonoid prevents the nephrotoxicity of BPA in rats, causing mitochondrial dysfunction due to ROS generation. In the kidney, BPA lowers the GSH levels and reduces CAT activity, increasing the formation of mitochondrial ROS and causing damage to the mitochondrial membrane. Quercetin restores the antioxidant parameters (GSH and MDA) in kidney tissues, suggesting that renoprotection is mediated by an antioxidant mechanism [161]. It also increases the activity of ATPase and SDH in the tissues of mice [65,162].

The effect of BPA on human hemoglobin is unclear, but the affinity between BPA and hemoglobin and erythrocytes is indicated. BPA has been observed to cause an increase in lipid peroxidation (LPO) in human erythrocytes and a decrease in SOD, CAT, and GPx activity. In a study by Sangai et al. [163], quercetin (10–50 µg/mL) was found to attenuate the toxic effects of BPA (150 µg/mL) in vitro in human erythrocytes. The beneficial effect of quercetin was mainly due to its detoxifying, nucleophilic, and free radical scavenging properties. Quercetin-supplementation may inhibit hemoglobin oxidation in the Fenton pathway [153].

### 5.8. Taurine

Taurine (2-aminoethanesulfonic acid) is a non-protein sulfur-containing amino acid, which is essential in the nutrition of children (especially premature babies) and a conditionally essential amino acid for adults. In humans, significant amounts of taurine are found in skeletal muscle, the heart, and the placenta. It plays an important role in human physiology, being the main antioxidant, anti-inflammatory, and anti-apoptotic factor in the body. It is a physiological stabilizer of cell membranes and a key element of the nerve and muscle conduction network. Taurine protects neurons from ROS or metal toxicity by maintaining the integrity of plasma membranes and organelles (especially mitochondria) of the cell. Taurine participates in the conjugation of bile acids to form bile salts in the liver, which facilitate the intestinal absorption of dietary lipids (including fat-soluble vitamins) [164].

In studies by Uzunhisarcikli et al., rats were administered taurine (100 mg/kg bw) with BPA (130 mg/kg). A protective effect of taurine against BPA-induced oxidative stress was observed, which can be attributed to its ability to scavenge ROS and induce antioxidant enzyme activity by activating the Nrf2 signaling pathway, similar to curcumin [75].

## 6. Conclusions

All over the world, BPA is still widely used in the production of plastics which are intermediates in the sourcing of everyday objects. The negative effects of this chemical compound have been extensively researched in recent years, resulting in many review articles. The conducted research shows that exposure to BPA induces the occurrence of organ pathology, which may ultimately contribute to the development of neoplasms. Undoubtedly, BPA disrupts the endocrine system. In addition, it can also negatively affect the organism’s oxidative homeostasis, as it not only causes increased ROS production and thus disrupts the redox balance, but also causes mitochondrial dysfunction and the modulation of cell signaling pathways. However, the influence of BPA on the process of carcinogenesis is still unclear and requires better understanding, especially in particular types of cancer. It is also necessary to conduct in vivo analyses on people that are professionally exposed to BPA in terms of cancer induction, as well as epigenetic research in this area. Further work is also required to determine the long-term impact of BPA-induced micro-RNA modifications on the neoplastic process. Also, research on the molecular mechanism of BPA’s action on the human body requires a more holistic approach, e.g., using the latest metabolomics tools. It is worth emphasizing that BPA toxicity may be exacerbated by a poor diet, metabolic disorders, and coexisting diseases. So far, no cheap alternative to BPA has been found, and the so far known BPA substitutes such as bisphenol F and bisphenol S are not safe alternatives at all. For this reason, ways to reduce the toxicity of BPA are intensively sought. One of the promising solutions seems to be the use of antioxidants with proven anti-cancer activity, such as genistein, curcumin, quercetin, naringin, and melatonin, which were discussed in this paper. The aforementioned polyphenols have also shown promising anti-cancer activity by targeting oncogenic and tumor suppressor micro-RNAs [165]. It should be emphasized, however, that although the results so far are promising, further research is needed to determine the optimal dosing of antioxidants. This is due, in part, to the fact that in many cases the positive effect of antioxidants in in vitro studies has not been confirmed by clinical studies. Additionally, self-performed uncontrolled antioxidant supplementation may have a negative impact on human health.

## Data Availability

Data is contained within the review.
